# The Diagnosis and Treatment of Diseases of the Ear

**Published:** 1886-03

**Authors:** 


**The Diagnosis and Treatment of Diseases of the Ear.** By OWEN D. POMEROY, M. D., Surgeon to the Manhattan Eye and Ear Hospital; Opthalmic and Aural Surgeon to the New York Infant Asylum; Consulting Surgeon to the Paterson Eye and Ear Infirmary, etc., etc. Second edition; revised, with additions; with one hundred illustrations. New York: D. Appleton & Co. 1886.

The first edition of this work was well received by the profession, and the second and enlarged will be gladly welcomed by those who had found the first of great value to them. This presents a much finer appearance, and much of the subject matter has been rewritten and additions made. Dr. Pomeroy intended this work for the use of the practitioner, and hoped it would prove valuable to some of the younger Aural Surgeons. Undoubtedly it has filled the place intended for it, and has been of great service to both specialist and physician. The second and somewhat larger work will be of greater service. It is comprehensive and complete. The illustrations from pen drawings, by Dr. J. O. Tansley, of New York, are clear and well executed. The typography and general character of the work is excellent, and up to the standard of this well-known publishing house.

